# Wheat glutamine synthetase *TaGSr-4B* is a candidate gene for a QTL of thousand grain weight on chromosome 4B

**DOI:** 10.1007/s00122-022-04118-8

**Published:** 2022-05-19

**Authors:** Fan Yang, Jingjuan Zhang, Yun Zhao, Qier Liu, Shahidul Islam, Wuyun Yang, Wujun Ma

**Affiliations:** 1 Australian-China Joint Centre for Wheat Improvement, Food Futures Institute, College of Science, Health, Engineering and Education, Murdoch University, 90 South Street, Murdoch, WA 6150 Australia; 2 Crop Research Institute, Sichuan Academy of Agricultural Sciences, 4 Shizishan Road, Chengdu, 610066 China; 3 College of Agronomy, Qingdao Agriculture University, Qingdao, 266109 China

## Abstract

**Key message:**

Glutamine synthetase* TaGSr-4B* is a candidate gene for a QTL of thousand grain weight on 4B, and the gene marker is ready for wheat breeding.

**Abstract:**

A QTL for thousand grain weight (TGW) in wheat was previously mapped on chromosome 4B in a DH population of Westonia × Kauz. For identifying the candidate genes of the QTL, wheat 90 K SNP array was used to saturate the existing linkage map, and four field trials plus one glasshouse experiment over five locations were conducted to refine the QTL. Three nitrogen levels were applied to two of those field trials, resulting in a TGW phenotype data set from nine environments. A robust TGW QTL cluster including 773 genes was detected in six environments with the highest LOD value of 13.4. Based on differentiate gene expression within the QTL cluster in an RNAseq data of Westonia and Kauz during grain filling, a glutamine synthesis gene (*GS*: *TaGSr-4B*) was selected as a potential candidate gene for the QTL. A SNP on the promoter region between Westonia and Kauz was used to develop a cleaved amplified polymorphic marker for *TaGSr-4B* gene mapping and QTL reanalysing. As results, TGW QTL appeared in seven environments, and in four out of seven environments, the TGW QTL were localized on the *TaGSr-4B* locus and showed significant contributions to the phenotype. Based on the marker, two allele groups of Westonia and Kauz formed showed significant differences on TGW in eight environments. In agreement with the roles of *GS* genes on nitrogen and carbon remobilizations, *TaGSr-4B* is likely the candidate gene of the TGW QTL on 4B and the *TaGSr-4B* gene marker is ready for wheat breeding.

**Supplementary Information:**

The online version contains supplementary material available at 10.1007/s00122-022-04118-8.

## Introduction

Wheat (*Triticum aestivum*) is one of the most important cereal crops and supplies food for 40% of human population (Pang et al. 2020). Due to the world's growing population, a high increase in wheat yield is required (Shiferaw et al. 2013). In general, wheat grain yield components include thousand grain weight (TGW), grain number per spike (GN) and spike number per area (Brinton and Uauy 2019). Among those, TGW showed high heritability, indicating the possibility of trait enhancement via genetic manipulation (Zhang et al. 2021). Several TGW QTL have been identified and potential candidate genes were revealed (Mohler et al. 2016; Xu et al. 2019; Guan et al. 2020; Wen et al. 2022).

In our recent study, 11 TGW QTL were detected on 1A, 1B, 4A, 4B, 4D, 5A, 5B, 7A, and 7B. Except 4D, 5A, 7A and 7B, all other QTL were detected in multiple field conditions or replicates (Zhang et al. 2021). In the literature, the TGW QTL was also reported on 1A (Arif et al. 2021), 1BL (Ren et al. 2021), 2DL (Li et al. 2021), 3A (Arif et al. 2021), 3D (Cui et al. 2014), 4A (Guan et al. 2018), 4BS (Guan et al. 2018; Xu et al. 2019; Li et al. 2021), 4BL (Guan et al. 2018; Xiong et al. 2021), 4D (Li et al. 2018), 5AL (Li et al. 2021), 5B (Yang et al. 2020), 5D (Li et al. 2018), 6A and 6B (Li et al. 2018; Arif et al. 2021), 6D (Cui et al. 2014), 7A and 7B (Guan et al. 2018; Li et al. 2018; Arif et al. 2021; Corsi et al. 2021), and 7D (Chen et al. 2020).

In addition, a number of genes for the seed size and TGW were identified, such as 1) a nuclear-encoded chloroplast protein gene *Tabas1-B1* (*Tabas1-B1a* and *Tabas1-B1b*) on 2BL (Zhu et al. 2016); 2) grain size related gene—*TaGS5-3A* (Ma et al. 2016), *TaGS-D1* (7D: 6.4 Mb bp) (Zhang et al. 2014b), *TaTGW6-4AL* (Hu et al. 2016a), and *TaTGW-7A* (Hu et al. 2016b); 3) a stress association protein (SAP) gene—*TaSAP1-A1* (7A: 585.0 Mb) and *TaSAP1-7B* (7B: 544.4 Mb) (Chang et al. 2013).

Previous studies have demonstrated that the grain size and grain yield were directly influenced by the levels of stem water soluble carbohydrate (WSC) and the WSC remobilization efficiency (Dreccer et al. 2009; Mir et al. 2012). The stem WSC levels before anthesis were associated with 57 and 74% of the grain yield of wheat and barley, respectively (Gallagher et al. 1976). On the stem WSC remobilization pathway, a fructan 1-exohydrolase (*1-FEH w3*) allele on 6B contributed to high TGW under drought conditions (Zhang et al. 2015).

A carbon consumer glutamine synthetase (GS) is considered as a connection point between the organic and inorganic phases, and a regulator between nitrogen and the carbon cycles in the chloroplast (Németh et al. 2018). The increasing amounts of carbon skeleton additional subunits lead to an increasing efficiency of GS2 oligomer (Németh et al. 2018). The expression levels of *GS* were regulated by the levels of nitrogen (N) and carbon (C) metabolites, in particular, controlled by the relative abundance of carbon skeletons (Oliveira and Coruzzi 1999). In their study, the transcription of *GS1* and *GS2* in cytosolic and chloroplast, respectively, was mediated and induced by light and sucrose level. The level of sucrose also changes the level of GS enzyme activity. GS in wheat was classified into four families: TaGS1, TaGS2, TaGSr and TaGSe (Bernard et al. 2008; Yin et al. 2022). Based on their subcellular locations, TaGS1, TaGSr and TaGSe belong to the cytosolic isoform while TaGS2 in the plastidic isoform (Swarbreck et al. 2011). During the leaf senescence in grain filling stage, the cytosolic isoform (GS1 and GSr) were predominant which suggested that they were critical for nitrogen remobilization to grain (Bernard et al. 2008). According to phylogenetic tree analysis of four GS clusters of GS1;1, GS1;2, GS1;3 and GS2, TaGS1 was classified to cluster GS1;1, TaGSr to GS1;2, TaGSe to GS1;3, and TaGS2 to GS2 (Thomsen et al. 2014). In addition to its function in ammonia assimilation, GS is critical for high grain production (Németh et al. 2018). In barley, the grain yield and nitrogen use efficiency (NUE) were improved through the over expression of *HvGS1;1* (Gao et al. 2019). In wheat, the grain number per spike and TGW were increased with the high *TaGS2-2Ab* gene expression level in the transgenic lines (Hu et al. 2018). Yu et al. (2021) found that sulphur regulates the GS activity in wheat leaves to increase NUE and grain yield (Yu et al. 2021). *TaGS2-A1*, *B1* and *D1* were mapped on group 2 and *GS-2B* were suggested as a candidate gene for grain protein content (GPC) QTL on 2B (Li et al. 2011; Nigro et al. 2020). *TaGS1* was mapped in group 6 chromosomes, while *TaGSr* and *TaGSe* were in group 4 (Bernard et al. 2008; Nigro et al. 2019; Wang et al. 2020).

The TGW QTL on 4B have been particularly highlighted since those are close to or overlapped with one of the green revolution genes—*Rht1* gene (Duan et al. 2020; Guan et al. 2020; Wen et al. 2022). However, the TGW QTL in the population of Westonia × Kauz was localized beyond the *Rht1* gene (Zhang et al. 2013). The QTL-hot spot for TGW was recently dissected into two QTL clusters (4B.1 and 4B.2) (Guan et al. 2020). The 4B.1 QTL was considered as the pleiotropic effects of *Rht1,* while the 4B.2 QTL seemed to form later in the grain filling stage although the candidate genes were not clear. Identifying the candidate genes of TGW QTL on 4B tends to be sophisticated but rather important for wheat yield improvement through breeding. The aim of the current study is to identify the candidate genes for TGW QTL on 4B.

## Materials and methods

### Plant materials

A wheat doubled haploid (DH) population of Westonia × Kauz with 225 DH lines were used for the TGW candidate gene identification. Westonia is a high yielding wheat cultivar bred in Western Australia. Kauz is a high yielding variety from the International Maize and Wheat Improvement Center (CIMMYT, EI Batan, Mexico) (Butler et al. 2005) and is drought tolerant (Rajaram et al. 2002). Both varieties achieve high yield with distinctive yield components, for example, the TGW in Westonia was significantly higher than Kauz, while the seed number per spike of Kauz is consistently higher than Westonia (Zhang et al. 2013). Kauz carries dwarfing gene—*Rht1* (*Rht-B1b* and *Rht-D1a*) (Butler et al. 2005), while Westonia carries *Rht2* gene (*Rht-B1a* and *Rht-D1b*) (Ellis et al. 2002; Botwright et al. 2005; Zhang et al. 2013). Those *Rht* genes were segregated in the Westonia × Kauz population and a TGW QTL was detected beyond the *Rht1* gene locus (Zhang et al. 2013).

### Glasshouse and field experiments

The previous TGW data of the 225 DH lines were included, which were from a glasshouse experiment at Murdoch University (Perth) in 2010, field experiments in Katanning in 2010 and Shenton Park in 2011. In glasshouse, three plants per line were grown in 4 L free-draining pots filled with potting mix prepared according to Zhang et al. (Zhang et al. 2009). The potting mix soil with sufficient fertilizer in each pot was weighed and pasteurized before planting. The pots were well-watered using mats underneath the pots and the pots were rotated on alternate days for receiving equal radiation exposure.

In Katanning (33.7° S, 117.6° E), due to the seed availability, the parental and 220 DH lines were grown under rain-fed condition and each line was in a separate 4.5 m^2^ (1.5 × 3 m) plot. In 2010, the annual rain fall in Katanning was low as 246 mm while the average was 437 mm from 1999 to 2012. In particular, during the grain filling period (August–October, 2010), the total rain fall was 27 mm while the average was 135 mm. Crops were managed with adequate nutrition, and spraying of herbicides and pesticides to control weeds and leaf diseases, respectively. In 2011, the parental and 223 DH lines were grown under adequate nutrition and water level (Zhang 2008) at Shenton Park field station (Perth, 32.0° S 115.9° E). The plot size for each line was 1.2 m^2^ (1.5 × 0.8 m). No spray was applied, and weeds were manually removed.

Plant height of three individual or representative (field) plants was measured at maturity. In glasshouse, all spikes (3) of each plant were taken for GN and TGW measurements while 20 and 3 random spikes from each DH line were taken in Shenton Park and Katanning field, respectively. The GN per spike and TGW were determined as grain parameters.

In 2016, two field experiments were carried out in Shenton Park field station and Wongan Hills (30.9° S, 116.7° E) (Zhao 2019). Complete randomised block designs were used for the two field sites. In Shenton Park, two parental lines and 152 DH lines were chosen for trials based on the seed availability. In addition to the same level base fertilizer, three nitrogen treatments (0, 50, 100 kg/Ha) were applied. Half of the 152 DH lines (71 lines) were replicated for final spatial adjustment. The individual plot size was 0.67 m^2^ (1 × 0.67 m). The nitrogen fertilizer was applied at the stem elongation stage. In Wongan Hills, the same nitrogen treatments (0, 50, 100 kg/Ha) were applied to the field trial. A group of 143 DH lines and the two parental lines were grown with individual plot size of 1.2 m^2^ (2 × 0.6 m). The nitrogen rate was used as a blocking factor in the design for those two field trials. The pesticides were sprayed in both stations and weeds were removed manually. During harvesting, five uniform plants from each plot were selected for phenotyping the yield components. For QTL analysis, each nitrogen rate was treated as one field condition, meaning a total of six field conditions in both field sites were evaluated.

### Linkage map construction

The 90 K SNPs were used to enrich the existing genetic linkage map that was constructed using SSR, 9 K SNPs and allele specific markers of *Rht-B1b*, *Rht-D1b*, *Vrn-A1a*, *Vrn-B1a*, and *Vrn-D1a* (Zhang et al. 2014a), etc. The SNP markers with large missing values of genotyping (> 25%) were excluded together with the distorted markers and double-cross markers. Most co-segregating markers were made redundant and removed from the genetic map. A fine map of Westonia and Kauz (WK) population was constructed with 3798 markers using Map Manager (Manly et al. 2001) and the QTL mapping package R/qtl (Broman and Sen 2009).

### QTL mapping

Inclusive composite interval mapping (ICIM) (IciMapping V4.1; http://www.isbreeding.net) was used for QTL detection (Li et al. 2015). The inclusive composite interval mapping addition (ICIM-ADD) method was selected for QTL mapping (Li et al. 2007). The mean value of replicates of individual DH line in each environment was used for QTL detection under a LOD score of 2.5. Permutations were set to 1000 at a significant level of 0.05.

### RNAseq analysis

A field drought experiment was carried out in 2011 (Zhang et al. 2015). The main stems (sheath included) were used for RNA extraction and later RNAseq analysis (Zhang et al. 2015; Al-Sheikh Ahmed et al. 2018). Samples collected between two to five weeks after anthesis at weekly interval were used for the RNAseq data analysis for this study. Three biological replicates of RNA extracts in each treatment (drought and irrigated) and growth stages were used for cDNA library construction with the Illumina TruSeq RNA v2 protocol and then sequenced using the Illumina HiSeq platform (100 bp paired end reads) using standard protocols at the Australian Genome Research Facility. The sequence data were obtained through the Illumina CASAVA1.8 pipeline. The data quality was checked by FastQC, and the low-quality reads were trimmed. Using Biokanga (version 3.4.2), those sequences were then aligned to the complete CDS contigs from the wheat sequencing consortium (https://www.wheatgenome.org/). Different gene expression analysis was conducted using DEseq (version 1.12.1) (Anders and Huber 2010) and edgeR (version 3.2.4) (Robinson et al. 2009). Gene expression level was normalized using model-based scaling normalization or Trimmed Mean of *M*-values (TMM).

### Database searches and sequence analysis

The gene sequences are available from the National Centre for Biotechnology Information (NCBI) (http://www.ncbi.nlm.nih.gov) and Ensembl Plants (https://plants.ensembl.org). These websites were used for a query to obtain closely related wheat genome sequences. The International Wheat Genome Sequencing Consortium (IWGSC) Survey Sequence Repository was used for exploring the upstream and downstream regions of the respective genes. The retrieved DNA sequences were analysed using CLC genomics workbench 10 and Geneious V.7.1.9.

### Primer design, PCR amplification of genomic DNA, cloning and enzyme digestion

Primer sequences were designed based on the reference sequence from IWGSC. The PCR amplification and analyses followed the previous protocol (Zhang et al. 2008). PCR products were centrifuged down at a high speed (14,000 RPM) in a filter tube and sequenced directly (ABI 377, Applied Biosystems). The enzyme digestion was performed in a total reaction volume of 20 μl that included 10 μl of PCR products, 10 units of enzyme (1 μl), and 1× rCutSmart^TM^ buffer (2 μl). The mixture was incubated at 37 °C overnight. The digested PCR products were run in a 1.5% agarose gel.

### Statistical analysis

Phenotypic and gene expression data were analysed by multivariate analysis of variance (MANOVA) using the general linear model implemented in IBM SPSS statistics 24 (https://www.ibm.com/au-en/products/spss-statistics). Post hoc Tukey’s Multiple Range tests were used to identify significant groupings. T test was used for the significance of parental phenotypes and different genotypic allele groups. The box and violin plots were generated through the ggplot2 package in R. The broad-sense heritability was calculated through Package lme4 in R-studio.

## Results

### Phenotypic variations

**Fig. 1:**
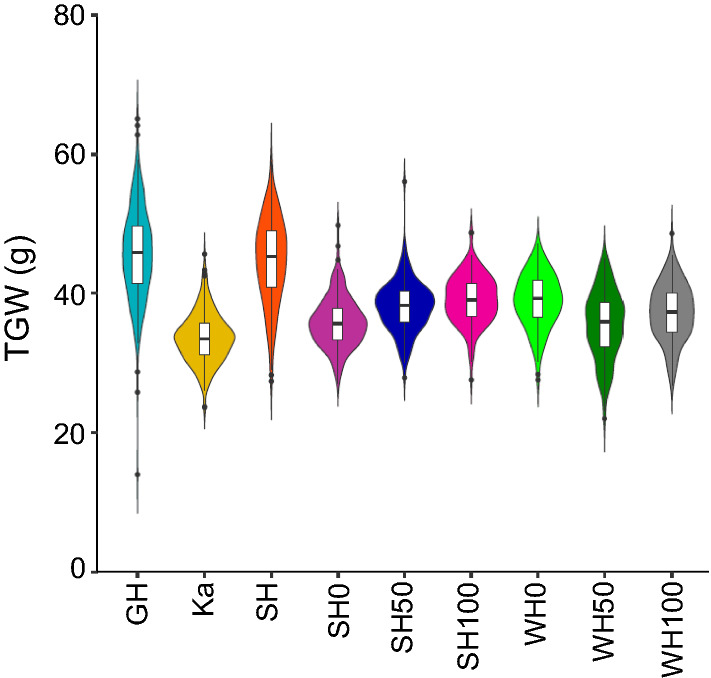
The distribution of thousand grain weight (TGW) in the DH population of Westonia and Kauz in nine environments. GH: glasshouse in 2010; Ka: Katanning in 2010; SH: Shenton Park in 2011; SH0, SH50, SH100: Nitrogen (N) level 0, 50 and 100 kg/ha in Shenton Park in 2016; WH0, WH50, WH100: N level 0, 50 and 100 kg/ha in Wongan Hills in 2016

Wheat cultivars of Westonia and Kauz in past studies consistently showed significant differences in grain yield components, such as Westonia with significantly higher TGW, while Kauz with significantly higher GN (Zhang et al. 2013) (Supplementary Table S1). The plant height of Westonia was significantly higher than Kauz in glasshouse. Since the TGW QTL with high *F*-value (19.7–37.9) were detected on 4B and strongly linked with the *Rht1* gene locus, further TGW QTL analysis was performed within this study for candidate gene identification. Apart from the previous data obtained in glasshouse and Katanning field station in 2010, and Shenton Park in 2011, a subset of 152 and 143 DH lines were planted in two field trials of Shenton Park and Wongan Hills in 2016, respectively. On average, the TGW was lower in comparison with previous data in glasshouse and Shenton Park, slightly higher than that from Katanning (Fig. 1). In 2016, slight differences were appeared between different nitrogen levels. In Shenton Park, TGW increased along with the N levels, while in Wongan Hills, it presented a different trend, which may be due to the different soil conditions. Overall, all TGW data showed normal distributions (Fig. 1 and Table S2). The TGW in nine environments showed the highest heritability (0.88) in comparison with the other phenotypes. In parallel, the TGW associated phenotypes of plant height, grain weight (GW), grain number per spike in glasshouse, Katanning (2010) and Shenton Park (2011) also showed normal distributions (Fig. S1).

### Genetic map of WK population

A fine map was constructed for QTL analysis. A total of 3798 markers formed 2651 bin markers and were allocated into 21 linkage groups with the total genome coverage of 7423.4 cM and an average bin marker interval of 2.8 cM. The total map length for each chromosome ranged from 202.1 cM (6D) to 617.6 cM (7A) and the minimum average bin marker interval was on 2B (1.7 cM) and maximum on 7D (12.4 cM) (Table S3).

### TGW QTL on 4B

**Fig. 2:**
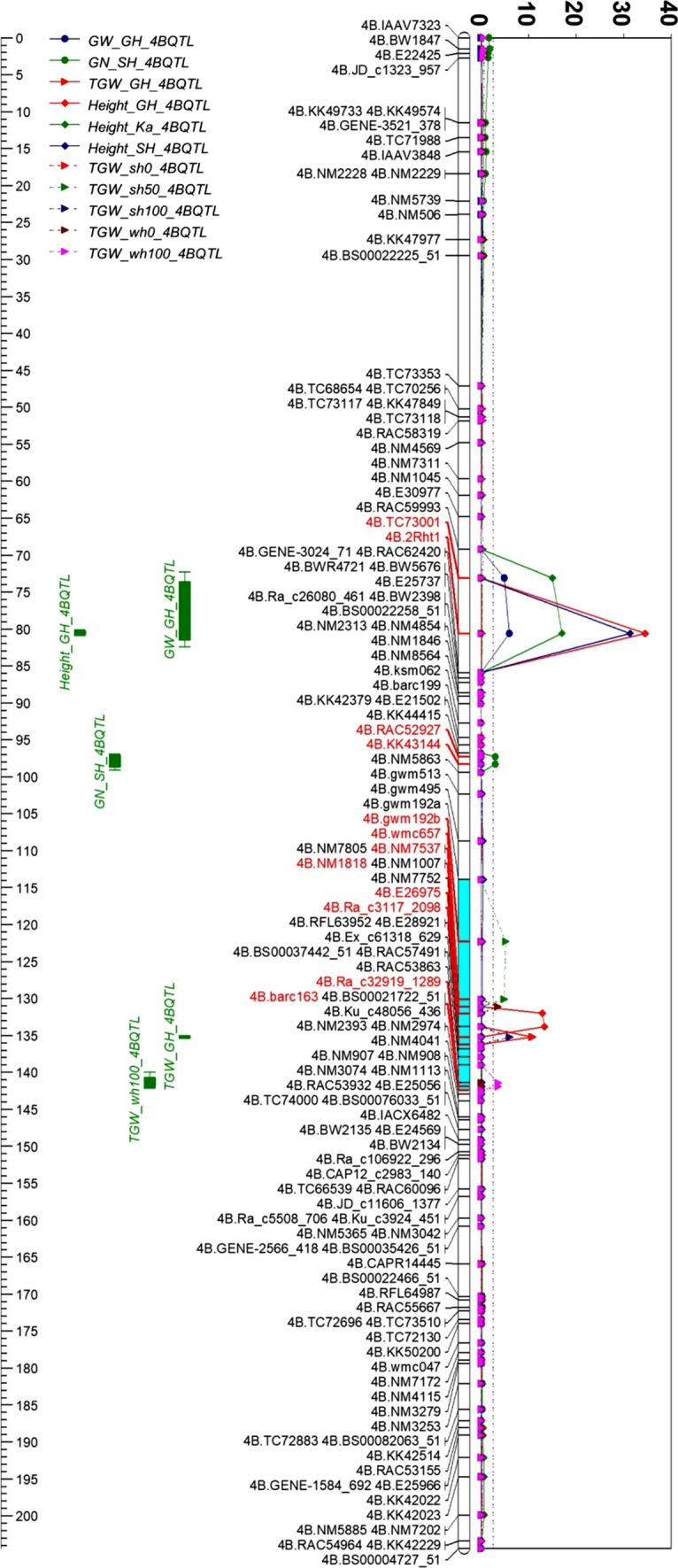
The QTL of thousand grain weight (TGW) detected on 4B in six environments together with the QTL of grain weight (GW) per spike, grain number (GN) per spike, plant height (Height) in glasshouse, Katanning, and Shenton Park. A TGW QTL cluster highlighted in light blue. GH: glasshouse in 2010; Ka: Katanning in 2010; SH: Shenton Park in 2011; sh0, sh50, sh100: Nitrogen (N) level 0, 50 and 100 kg/ha in Shenton Park in 2016; wh0, wh100: N level 0 and 100 kg/ha in Wongan Hills in 2016

A TGW QTL cluster was detected with three QTL linked together on positions 125–133, 135–137 and 141–143 cM. The QTL cluster was 40 cM beyond *Rht1*. The positive effects of all QTL were contributed by Westonia with the highest LOD value of 13.4 and the largest PVE value of 21.2% on the position of 132–137 cM (Fig. 2 and Table S4).

The QTL for GW and plant height were on the *Rht1* gene region while a grain number (GN) QTL was at 24 cM beyond the *Rht1* locus and another plant height QTL was overlapped with the TGW QTL cluster (Fig. 2 and Table S4).

### Candidate genes within TGW QTL cluster on 4B

Within the TGW QTL cluster on 4B, there were 773 genes (*TraesCS4B02G236800–TraesCS4B02G371300*) between markers gwm192b and barc163 (4B: 460–580 Mb in the physical map) (Table S5). Since the grain weight was accumulated through the grain filling stage, and the stem WSC was heavily involved in grain filling, the stem RNAseq data between 11 and 31 days after anthesis (DAA) were used for candidate gene selection. Out of the 773 genes, 315 gene expression data were captured, and four genes showed two transcripts in Kauz but one in Westonia. Those genes were *TraesCS4B02G240100* (4B: 497.86 Mb), *TraesCS4B02G240900* (4B: 499.90 Mb), *TraesCS4B02G251800* (517.95 Mb), and *TraesCS4B02G262900* (532.72 Mb). The first gene—*TraesCS4B02G240100* was described as two-component response regulator in Ensembl Plants (ENBK-EBI) since it contained Myb (a member of myeloblastosis family) domain and a receiver domain—signal transduction response regulator. The second gene—*TraesCS4B02G240900* was a glutamine synthetase (*GS*) on 4B. *GS* is an important gene for nitrogen (NH_4_^+^) absorption and involved in glutamine synthase for protein formation in plants. The third gene—*TraesCS4B02G251800* is a putative Nodulin-related protein and belongs to transmembrane protein family (Denancé et al. 2014). Overexpression of the *Nodulin-related protein 1* decreased the thermotolerance through reducing the accumulation of abscisic acid (ABA) after heat shock treatment in *Arabidopsis* (Fu et al. 2010). The fourth gene—*TraesCS4B02G262900* is a putative mannitol transporter. The high gene expression levels and mannitol transport activity of mannitol transporter are parallel with the salt and drought-tolerant capacity in olive trees (Conde et al. 2011). The salt and drought levels significantly increased the mannitol transport activity and mannitol transporter gene expression level in wheat (Abebe et al. 2003).

**Fig. 3:**
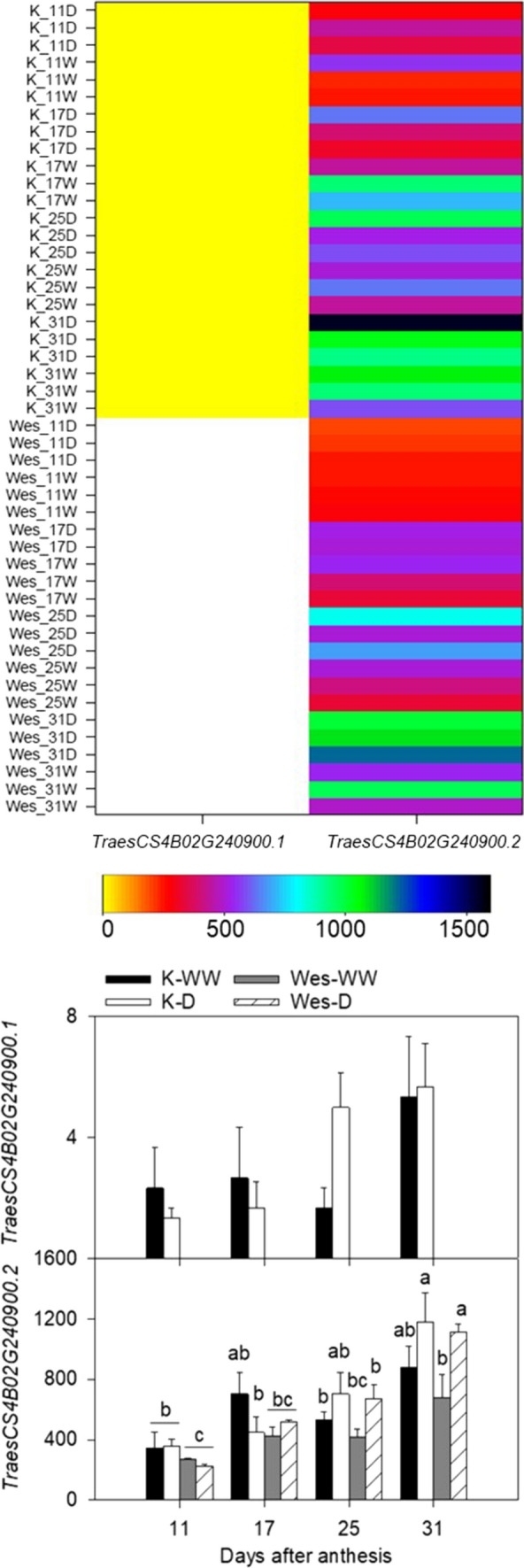
Different gene expression levels of two *TraesCS4B02G240900* transcripts between Westonia and Kauz in different growth stages (days after anthesis) and treatments. K: Kauz; Wes: Westonia; D: drought; W or WW: well-watered. The vertical bars represent SE; values with the same letter are not different at *p* < 0.05

From the comparisons of the gene expression levels, no significant differentiation in gene expression levels between Westonia and Kauz was detected on *TraesCS4B02G251800* (517.95 Mb) while the only difference showed between treatments at 25 DAA in Westonia on *TraesCS4B02G240100* (497.87 Mb) (Fig. S2 and Table S6). The gene expression of *TraesCS4B02G262900* gradually decreased after 17 DAA at irrigated condition in both varieties. The significantly lower gene expression levels were observed under drought relative to irrigated in both varieties after 17 DAA while high small sugar molecules (glucose, fructose and sucrose) significantly increased under drought (Zhang et al. 2015). In addition, no significant difference was presented between Westonia and Kauz in both treatments. Those results indicate that gene—*TraesCS4B02G262900* may not contribute significantly to the sugar remobilization. However, the gene expression of *TaGS-4B* (*TraesCS4B02G240900*, 499.90 Mb) gradually increased and was significantly enhanced under drought, especially in Westonia (Fig. 3). It tends to be a candidate gene for the TGW QTL.

### *TaGS-4B* gene expression levels

There were two *GS* transcripts in Kauz, while only one transcript showed in Westonia (Fig. 3 and Table S6). Low expression levels ranged 0–8 units were showed on *TraesCS4B02G240900.1* and no significant difference was identified between treatments. However, the expression levels on *TraesCS4B02G240900.2* ranged 95–1563 units and displayed clear increasing patterns in both treatments and varieties along with the growth stages, especially under drought. At 31 DAA, the expression level was significantly higher under drought compared to the well-watered in Westonia, while the same pattern also appeared in Kauz but with non-significance (Fig. 3). The results indicate that GS may play a role in nitrogen remobilization efficiency under drought.

### *TaGS-4B* is *TaGSr1*

**Fig. 4:**
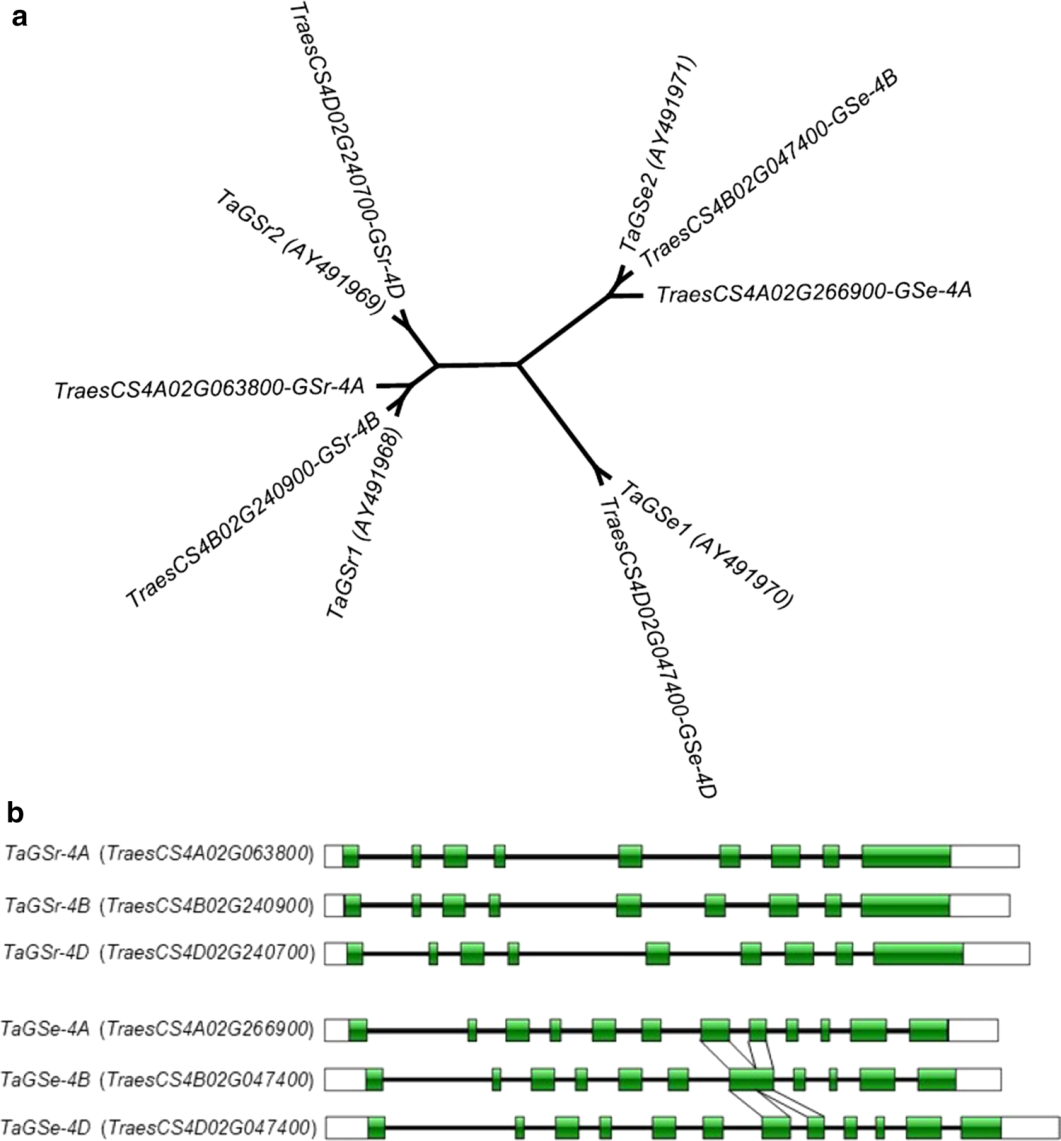
Unrooted phylogenetic tree of the DNA sequences of glutamine synthesis genes on 4B (**a**) and the gene structures of *TaGSr* and *TaGSe* (**b**). *TaGSr-4B* is identical to *TaGSr1*

It was reported that there were four groups of *GS* in wheat, including *TaGS1*, *TaGS2*, *TaGSr* and *TaGSe* with three copies of *TaGS1* and *TaGS2*, two copies of both *TaGSr* and *TaGSe* while both *TaGSr* and *TaGSe* were on group 4 chromosomes (Bernard et al. 2008). The accession IDs of AY491968 (*TaGSr1*), AY491969 (*TaGSr2*), AY491970 (*TaGSe1*) and AY491971 (*TaGSe2*) in NCBI were used to blast against the wheat genome. *TaGS-4B* (*TraesCS4B02G240900*) showed 100% identity with *TaGSr1,* while *TaGS-4D* (*TraesCS4D02G240700*) was identical to *TaGSr2* (Fig. 4a). Another homologous gene on 4A was identified as *TraesCS4A02G063800*. All those *TaGSr* genes have the exact same size of nine exons and eight introns. For clear identification, those *TaGSr* genes have been named as *TaGSr-4A*, *TaGSr-4B* and *TaGSr-4D* (Fig. 4b). Similarly, three homologous genes of *TaGSe* were also identified in group 4 chromosomes, including *TraesCS4B02G047400* and *TraesCS4D02G047400* standing for *TaGSe1* and *TaGSe2*, and the later—*TraesCS4A02G266900* unidentified before (Fig. 4a). For clear identification, those *TaGSe* genes have been named as *TaGSe-4A*, *4B* and *4D*. *TaGSe-4A* and *4D* showed 12 exons and 11 introns, while *TaGSe-4B* had one less exon and intron as the exon 7 and 8 were combined as exon 7 (Fig. 4b). *TaGSe-4B* and *TaGSr-4B* were located on the short and long arms on 4B, respectively.

### Polymorphisms on *TaGSr-4B*

**Fig. 5:**
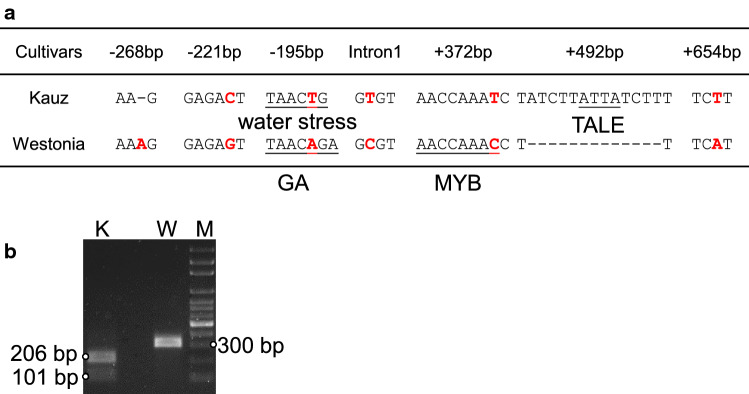
Gene sequence polymorphisms on *TaGSr-4B* between Westonia and Kauz. ** a** The one SNP insertion (− 268 bp) and two SNPs (− 221 bp and − 195 bp) in the promoter region, and two SNPs (+ 372 bp, + 654 bp) and a 13-bp deletions on the 3′ untranslated regions, and one SNP in intron 1 (the related four motifs were listed); ** b**. the cleaved amplified polymorphic (CAP) marker showed 102 bp difference between Westonia (308 bp) and Kauz (206 bp) using cutting enzyme –BcoDI (GTCTCN’/CAGAG) on − 221 bp in the promoter. *GA* gibberellin, *MYB* myeloblastosis, *TALE* transcription activator-like effector

From the gene expression analysis within the TGW QTL cluster, *TaGSr-4B* tends to be a candidate gene for the QTL. The *GS* gene sequences including the promoter regions and downstream untranslated regions (UTR) were isolated from Westonia and Kauz using primer pairs designed specific for 4B chromosome (Table S7 and Fig. S3). In the promoter region, one bp insertion on the position of 268 bp, two SNPs on the positions of 195 and 221 bp in Westonia ahead of the coding region were identified. One and two SNPs were detected on intron 1 and 3′ terminal UTR region, respectively. Moreover, a 13-bp deletion was revealed on the 3′ terminal UTR region in Westonia (Fig. 5a). Interestingly, all above SNPs, a 1-bp insertion and a 13-bp deletion occurred in Westonia only while the sequences of Kauz were the same as Chinese Spring.

Within the polymorphisms between parental lines, on the promoter region (− 221 bp), the sequence differences involved two different motifs including water stress motif—TAACTG in Kauz and gibberellin (GA) motif—TAACAGA in Westonia. On the 3′ terminal UTR region, the SNP mutation (+ 372 bp) in Westonia led to an additional motif of MYB while the 13-bp deletion in Westonia caused a motif—transcription activator-like effector (TALE) deleted.

**Fig. 6:**
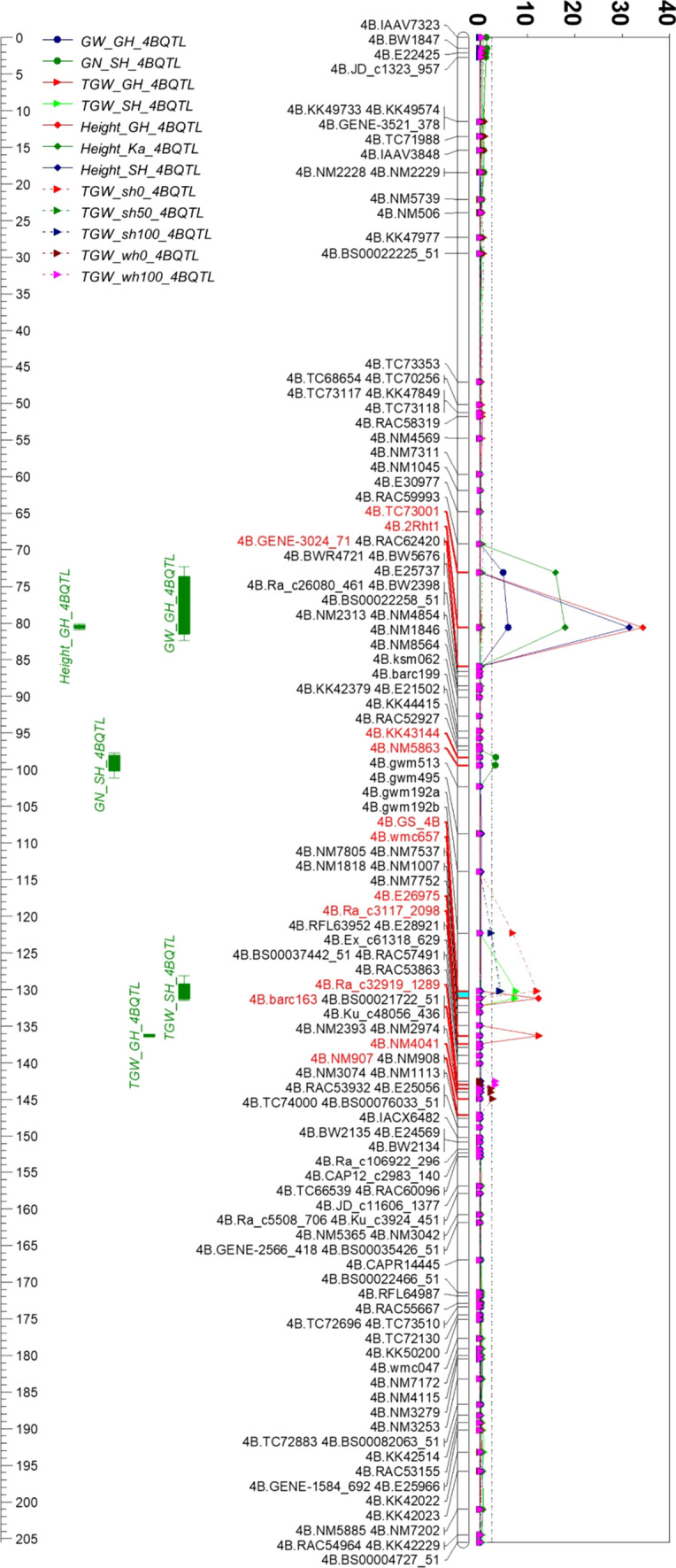
The QTL of thousand grain weight (TGW) detected on 4B in seven environments together with the QTL of grain weight (GW) per spike, grain number (GN) per spike, plant height (Height) in glasshouse, Katanning, and Shenton Park while *TaGSr-4B* gene marker (GS_4B) was included in the map. *TaGSr-4B* gene (GS_4B) was mapped above the SSR marker—WMC657, and the TGW QTL cluster highlighted in light blue were on/near the region of the gene marker GS_4B (*TaGSr-4B*). GH: glasshouse in 2010; Ka: Katanning in 2010; SH: Shenton Park in 2011; sh0, sh50, sh100: Nitrogen (N) level 0, 50 and 100 kg/ha in Shenton Park in 2016; wh0, wh100: N level 0 and 100 kg/ha in Wongan Hills in 2016

Based on those SNPs between Westonia and Kauz, a primer pair of En760F and En1045R was designed since the SNP on the position of − 221 bp aligned with the enzyme cutting sequence of BcoDI/BsmAI (GTCTCN’/CAGAG; detected with NEBcutter V2.0 software). The primer pair was used for amplifying a 308 bp fragment in both varieties. The fragment in Kauz was cut into two fragments of 101 and 206 bp, while it remained the same size in Westonia (308 bp). The 102 bp fragment difference between Westonia (308 bp) and Kauz (206 bp) was easily identified in a 1.5% agarose gel (Fig. 5b). This cleaved amplified polymorphic (CAP) marker was used for screening *TaGSr-4B* in the WK DH population, which was mapped above an SSR marker WMC657 (Fig. 6).

### New TGW QTL on 4B

The WK genetic map including *TaGSr-4B* CAP marker (GS_4B) was used for TGW QTL reanalysis on 4B. Interestingly, a TGW QTL was detected in one more environment—the Shenton Park field trial in 2010 (Shenton2010). Under four field conditions (TGW_SH, TGW_sh0, TGW_sh50, TGW_sh100), the TGW QTL were laid on the GS_4B marker region while no change was noticed on TGW QTL in glasshouse. The TGW QTL in N0 and N100 in Wangan Hills were moved to 10 and 7 cM below in comparison with the previous QTL, respectively (Fig. 6 and Table S8). The positive allele of all TGW QTL were from Westonia accounting for 8–25% of the phenotype variations.

Since the *Rht1* gene was reported to contribute to TGW (Guan et al. 2020), its role for TGW in the WK population was further analysed using plant height. The same results appeared that the QTL of plant height in three environments were on the *Rht1* gene region which was 40 cM above the TGW QTL cluster. It could conclude that the *Rht1* gene that confers the plant height may not contribute to the TGW QTL in the current population (Fig. 6). One height QTL (LOD: 12.4) attributed to Westonia was overlapped with the TGW QTL (Fig. 6 and Table S8), indicating the overall contribution of plant height to TGW (Wen et al. 2022).

### The CAP marker of *TaGSr-4B* correlates with superior TGW

**Fig. 7:**
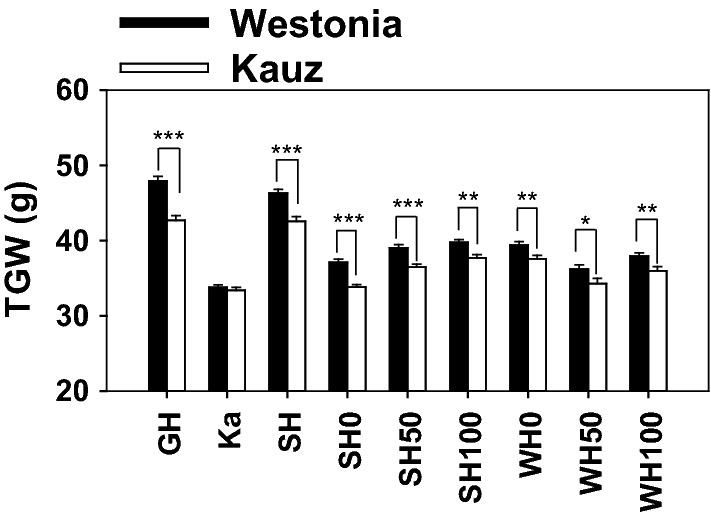
Significant differences on thousand grain weight between Westonia and Kauz allele types in DH population under eight environments. GH: glasshouse in 2010; Ka: Katanning in 2010; SH: Shenton Park in 2011; SH0, SH50, SH100: Nitrogen (N) level 0, 50 and 100 kg/ha in Shenton Park in 2016; WH0, WH50, WH100: Nitrogen (N) level 0, 50 and 100 kg/ha in Wongan Hills in 2016

The *TaGSr-4B* marker—GS_4B was used to separate the WK population into two allelic groups of Westonia and Kauz. Significant differences between the two groups were identified in TGW under eight environments. The Westonia type increased TGW by 5–12% in comparison with the Kauz type (Fig. 7).

## Discussion

### Effects of multiple transcripts

Genetic variants influencing RNA processing are similar to the variants affecting transcription. The sequence variations on pre-mRNA would impact the stability, translation and structure of mRNAs, causing phenotypic diversity (Gingeras 2007; Manning and Cooper 2017). Apart from the basal RNA processing, some genes have alternative splicing and express multiple transcripts, which may encode different isoforms. The major differential pro-mRNA processing is mediated in a specific tissue and stage, or in response to particular regulators (Manning and Cooper 2017). In this study, the genes within the TGW QTL cluster with multiple transcripts were chosen for their potential function for the phenotypes.

A total of four genes including *TaGSr-4B* within the TGW QTL cluster were detected with two transcripts in Kauz and one in Westonia. *TaGSr-4B* showed gradually increased gene expression patterns along with the growth stages. The gene expression levels of *TaGSr-4B* were almost in parallel with the sucrose and fructose level measured previously (Zhang et al. 2015). The significant differences in gene expression levels were presented between treatments in Westonia. In addition, *GS* gene was previously reported to be associated with TGW in Maize (Hirel et al. 2001; Martin et al. 2006) and wheat (Habash et al. 2001; Hu et al. 2018). Therefore, *TaGSr-4B* was chosen for this study.

### The associations between *GS* genes and TGW

Several grain yield-related QTL showed linkages with the localization of *GS*. For example, in maize, the QTL of grain yield and TGW were colocalized on the region of *GS* on chromosome 5 (Hirel et al. 2001). Strong positive correlations between grain yield and GS activity were also observed (Gallais and Hirel 2004). Further knockout *ZmGln1;3* and *ZmGln1;4*, the kernel number and kernel weight were reduced in the mutants (Martin et al. 2006). In rice, the QTL of one-spikelet weight was on the region of *OsGS1*, and the *OsGS1;1* knockout mutant showed a severe delay in grain filling under normal nitrogen level (Obara et al. 2001; Tabuchi et al. 2005). In barley, overexpression of *HvGS1;1* achieved higher grain yield and NUE compared with the wild-type under three nitrogen levels (Gao et al. 2019). In sorghum, the transgenic lines with higher levels of a sorghum cytosolic *GS* gene—*Gln1* transcripts exhibited a higher GS activity and showed 2.1-fold increased shoot biomass in relative to controls (Urriola and Rathore 2015). In wheat, the transgenic lines with favourable allele—*TaGS2-2Ab* increased GS activity and enhanced grain yield, grain number per spike and TGW (Hu et al. 2018). During senescence, the increased GS1 activity in the transgenic wheat lines enhanced the capacity of nitrogen accumulation in grain resulting in increased root and grain dry weight (Habash et al. 2001). Those results conclude that high gene expression levels of *GS* lead to increased GS activities which results in high grain yield and TGW. From our RNAseq results, the transcription levels of *TaGSr-4B* were gradually increased along with the growth stages. In particular, the gene expression levels were significantly higher under drought in Westonia, which evidence the higher remobilization during the late grain filling stage leading to the significantly high TGW.

In addition, the TGW QTL on 4B has been highlighted by a number of researchers (Li et al. 2018, 2021; Xu et al. 2019; Arif et al. 2021; Corsi et al. 2021). In their studies, the TGW QTL was overlapped or very close to the *Rht1* locus, or plant height QTL (Li et al. 2018; Xu et al. 2019; Guan et al. 2020; Wen et al. 2022). However, in our previous study using the population of Westonia and Kauz, the TGW QTL was located beyond the polymorphic *Rht1* locus (Zhang et al. 2013). So far, the map of Westonia and Kauz was enriched using 90 K SNPs. The bin markers on 4B have been increased from 28 to 87. The TGW QTL were clearly showing 40 cM beyond the *Rht1* locus. Therefore, it is unlikely to state that *Rht1* had the major contribution to the TGW QTL in the WK population although the association still exists due to the linkage on 4B. In fact, from the QTL and *TaGSr-4B* allele analysis, *TaGSr-4B* is likely the major candidate gene for the TGW QTL in the population of Westonia and Kauz. Nevertheless, a possibility still exists in that both *TaGSr-4B* and *Rht1* contribute to TGW but the *TaGSr-4B* has a much stronger effect that masks the *Rht1* effect in the WK population.

### GS family and function

Glutamine synthetase (GS; EC 6.3.1.2) is one of the main enzymes accumulating inorganic N through the ATP-dependent conversion from glutamine into glutamate (Yin et al. 2022). GS has been focused by multiple studies on nitrogen use efficiency in plants (Hirel et al. 2001; Zhang et al. 2017; Hu et al. 2018). High GS activities were correlated well with high grain yield due to its important role in N-remobilization (Gallais and Hirel 2004). High N-remobilization after flowering can also affect the grain filling leading to high grain weight (Gallais and Hirel 2004). A particular study on the interdependence of C and N metabolism in maize kernel development revealed that the supply of N assimilates facilitates kernel’s utilization of carbohydrates (Below et al. 2000). The expression levels of *GS* were controlled by the relative abundance of carbon skeletons, and the level of GS enzyme activity is regulated by the level of sucrose (Oliveira and Coruzzi 1999).

The sequences of *GS* isoforms include a range of light-responsive elements, auxin-responsive elements and binding sits for transcription factors such as MYB and Dof (Castro-Rodríguez et al. 2011). MYB transcription factor Circadian Clock-Associated 1 (CCA1) in Arabidopsis was predicted to have a role in regulating *AtGln1;3* expression under the availability of C skeletons (Gutiérrez et al. 2008; Gutiérrez 2012). It was suggested that transcription factor—DNA-binding One Zinc Finger (Dof)1 was involved in the regulation of C4 photosynthetic phosphoenolpyruvate carboxylase (C4PEPC) gene expression, and Dof2 mediated multiple gene expressions associated with carbon metabolism pathway (Yanagisawa 2000, 2002).

From KEGG analysis, *TaGSr-4B* is involved in the glyoxylate and dicarboxylate metabolisms (path ko00630), energy metabolism (nitrogen metabolism, path: ko00910), amino acid metabolism (alanine, aspartate and glutamate metabolisms, path: ko00250), and arginine biosynthesis (path: ko00220). In addition, *TaGSr-4B* is involved in environmental signal transduction (two-component system, path: ko02020) and signaling and cellular processes (exosome, brite: ko04147), as well as in cell growth and death (necroptosis, path: ko04217). More importantly, the substrate-glutamate in glutamine synthesis is formed from alpha-ketoglutarate generated from sucrose in mitochondria. The metabolic pathways clearly indicates associations between the carbon levels and N assimilation. Taken together, *GS* gene expression is regulated by the available C skeleton and amino acid and involved in the interdependence of N and C metabolisms.

There have been two types of GS (GSI and GSII) identified in plants, with the genome sequence size of *GSI* larger than *GSII* (Swarbreck et al. 2011). The GSI is in the cytosol, while the GSII encoding the plastidic isoform is located and active in mainly chloroplast and merely mitochondria (Taira et al. 2004). In wheat, the cytosolic GSI have three subtypes (TaGS1, TaGSr and TaGSe). During the grain development, *TaGS1* and *TaGSr* were the predominant forms presented in root, flag leaf, peduncle and glume, in particular, *TaGSr* showed high gene expression in peduncle (Bernard et al. 2008). During leaf senescence and grain filling, the mesophyll cells disappeared, while the vascular cells containing TaGS1 (in the vascular tissue) and TaGSr (in the phloem sieve elements) remained intact, which further support the roles of assimilate remobilization (Bernard et al. 2008).

For the mRNA stability and translation efficiency, untranslated regions (UTRs) of genes are one of the crucial regulator (Rao et al. 2017). In comparison with the *TaGSr-4B* sequences of Westonia and Kauz, no difference was found in the coding region. However, two SNPs and a 1-bp insertion in Westonia were identified in 5′-UTR while two SNPs and a 13-bp deletion in Westonia in the 3′-UTR region. In addition, one SNP was identified in Intron 1. Particularly, at the position of − 195 bp, one motif (TAACTG) in Kauz was related to water stress, whereas the one (TAACAGA) in Westonia was a gibberellin (GA) responsive motif. The Kauz motif (TAACTG) found in the simian virus 40 enhancer and the maize bronze-1 promoter is a consensus MYB recognition sequence bound by *ATMYB2* (Urao et al. 1993), which was predicted as a transcriptional activator in ABA-inducible expression of a dehydration-responsive gene, *rd22*, therefore, involved in regulation of genes that are responsive to water stress in *Arabidopsis* (Abe et al. 1997). It was suggested that genes with a MYB transcription factor (TF) binding motif in their promoters were controlled by MYB TF, which was induced by ABA (Lewis et al. 2015). Coincidently, a GA responsive element was on the same promoter region. GA is a plant hormone in regulating of plant growth. The Westonia motif TAACAGA is a GA response complex which is conserved in the promoters of alpha-amylase and proteinases in cereals (Sutoh and Yamauchi 2003). In addition, there was a MYB binding site (AACCAAAC) fitted MYB binding motif MACCWAMC ([AC]ACCWA[AC]C) in the 3′-UTR region followed by a 13-bp deletion in the Westonia type *TaGSr-4B*. Within the 13-bp deletion, one motif of TALE was missed. The 3′-UTR region plays a crucial role in gene expression through influencing the mRNA localization, stability, export, and translation efficiency and also establishes protein–protein interactions (Mayr 2019). TALE transcription factors could adjust the gene expression and also be a N-terminal translocation signal or a signal for unique chromosome targeted (Morbitzer et al. 2010).

### The contributions of other genes

While the TGW QTL in the most environments were overlapped with the *TaGSr-4B* locus, the TGW QTL in glasshouse, and in N0 and N100 field trials in Wongan Hills are still 5 and 13 cM beyond the *TaGSr-4B* locus, respectively. Although the *TaGSr-4B* locus has been confirmed with the highest stringent in conferring the TGW phenotype, it is still possible that some other genes within the QTL also contribute to the phenotype. For further identifying other potential candidate genes, the entire gene expression profile on 4BL was drawn from the RNAseq data of Westonia and Kauz, and genes within the TGW QTL cluster (460–580 Mb) were investigated. Besides the multiple transcripts, the potential candidate gene selections followed the criteria: (1) the maximum expression level is over 100; (2) the average differentiation expression between Westonia and Kauz is over four times. As a result, two potential candidate genes were selected, including *TraesCS4B02G222300* (466.96 Mb) and *TraesCS4B02G225400* (471.96 Mb) (Fig. S4). The average expression levels of *TraesCS4B02G222300* were 12.7 and 161.1 in Kauz and Westonia, respectively, with the differentiation of 12.7-fold (Fig. S4). The gene function is related to the transcription initiation factor IIF, alpha subunit (TFIIF). TFIIF is a transcription regulator and promotes transcription elongation (Gaudet et al. 2011). The different expression levels between Westonia and Kauz may regulate the gene expression in the network. *TraesCS4B02G225400* is a heat shock protein with the average gene expression level of 130.3 in Westonia, whereas no gene expression was detected in Kauz (Fig. S4). Heat shock proteins are the key components responsible for protein folding, assembly, translocation, and degradation in normal cellular processes or under stress conditions (Park and Seo 2015). Further investigations are required for those potential candidate genes in contributing to the TGW QTL.

## Conclusion

TGW is one of the three components of grain yield in wheat. The candidate gene identification of TGW QTL on 4B is crucial for its application in wheat breeding. A TGW QTL with a high *F*-value (37.9) was identified in a large interval on 4B in the DH population of Westonia × Kauz. Further wheat 90 K SNP array was screened across the DH population, and a highly saturated map was generated. The TGW data of nine environments formed a robust TGW QTL cluster including 773 genes. Within the QTL cluster, the gene expression data were derived from a RNAseq data of Westonia and Kauz. After filtering the differential gene expression levels and multiple transcripts, *TaGSr-4B* was selected as a potential candidate gene for the TGW QTL. The sequence variations were identified between Westonia and Kauz, and a CAP marker was generated for the gene mapping and the TGW QTL reanalysing. Surprisingly, in four out of seven environments, the TGW QTL were localized on the *TaGSr-4B* locus and showed significant contributions to the phenotype. The marker was used to separate the Westonia and Kauz allele types. Significant allelic effects on TGW were detected in eight out of nine environments. In agreement with the roles of *GS* genes on nitrogen remobilization in parallel with the functions of carbon remobilization, *TaGSr-4B* is most likely the candidate gene of the TGW QTL on 4B in Westonia and Kauz population. The *TaGSr-4B* gene marker can be used in wheat breeding. Since there were three TGW QTL were mapped in slightly different locations, it cannot rule out that other genes may also contribute to TGW.

## Supplementary Information

Below is the link to the electronic supplementary material.

Supplementary file1 (PPTX 362 kb)

Supplementary file2 (XLSX 45 kb)

## Data Availability

Electronic supplementary material The online version of this article (https://doi.org/xxxxx) contains supplementary material, which is available to authorized users.
