# Supplementary material for: Wheat glutamine synthetase TaGSr-4B is a candidate gene for a QTL of thousand grain weight on chromosome 4B
Source: Theor Appl Genet. 2022 May 19;135(7):2369–84. doi: 10.1007/s00122-022-04118-8 (PMC9271121; doi:10.1007/s00122-022-04118-8)
Supplement: Supplementary file 1 — Supplementary file1 (PPTX 362 kb) [file 122_2022_4118_MOESM1_ESM.pptx]

## Slide 1
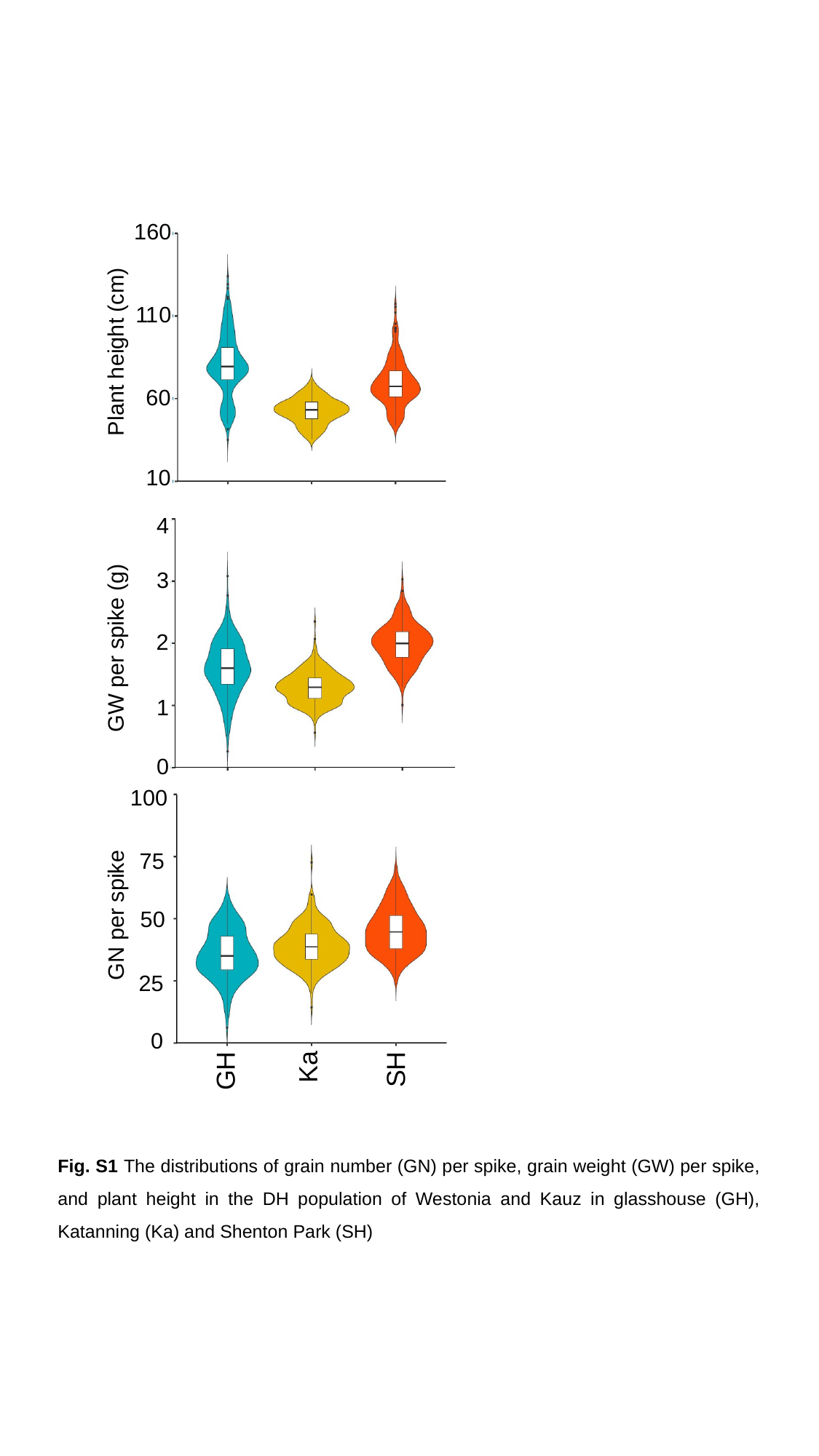

Fig. S1 The distributions of grain number (GN) per spike, grain weight (GW) per spike, and plant height in the DH population of Westonia and Kauz in glasshouse (GH), Katanning (Ka) and Shenton Park (SH)

## Slide 2
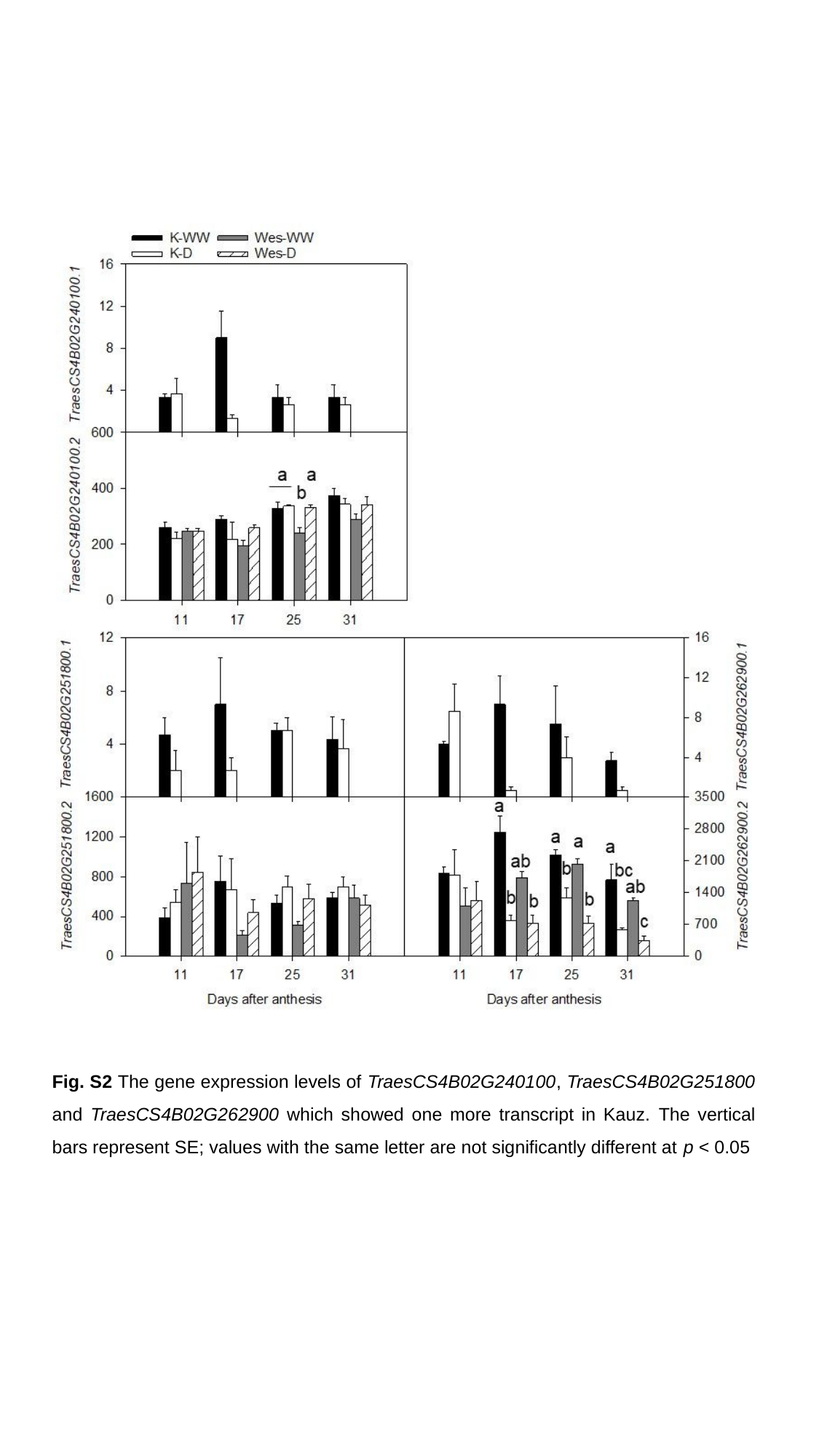

Fig. S2 The gene expression levels of TraesCS4B02G240100, TraesCS4B02G251800 and TraesCS4B02G262900 which showed one more transcript in Kauz. The vertical bars represent SE; values with the same letter are not significantly different at p < 0.05

## Slide 3
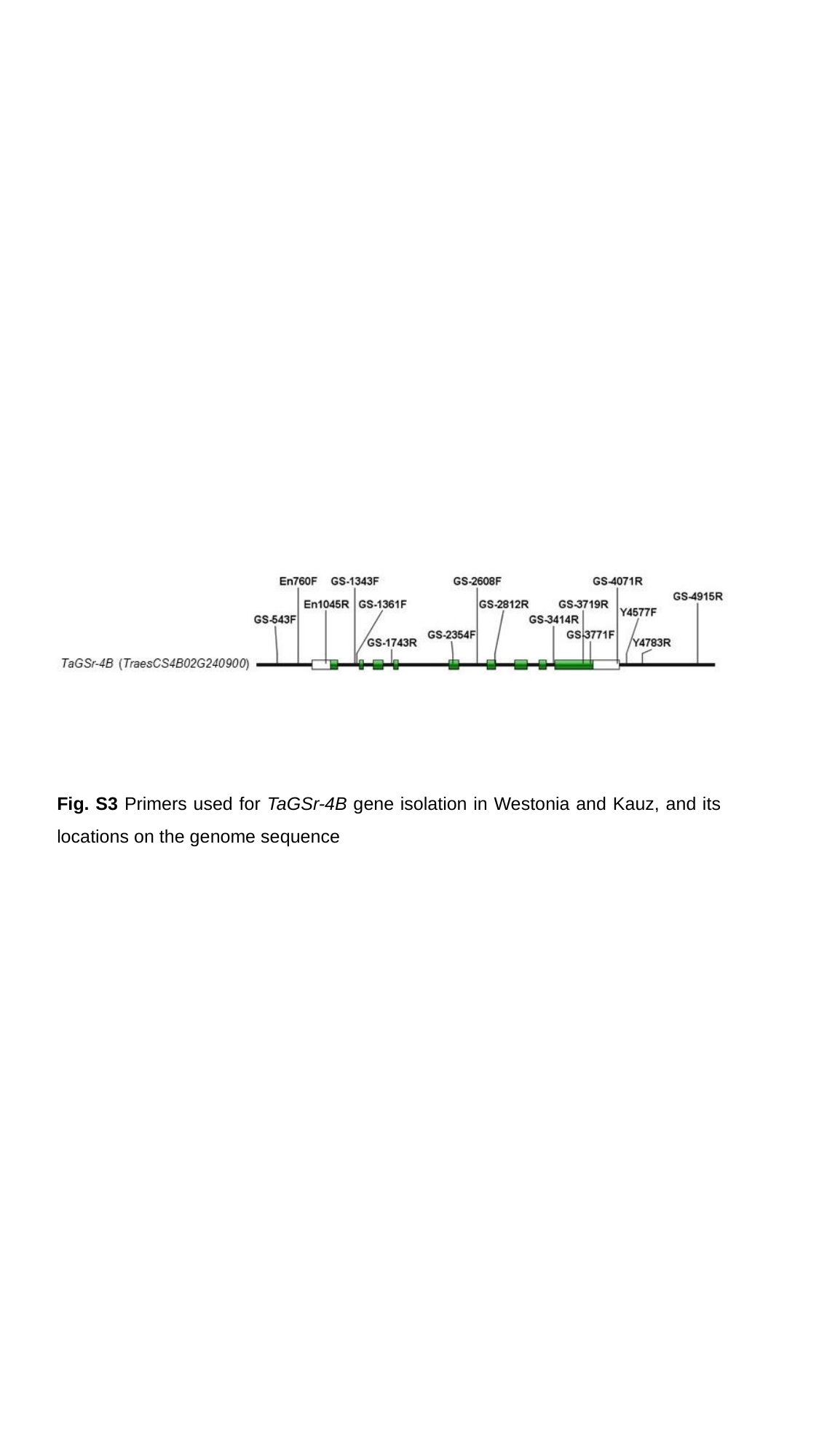

Fig. S3 Primers used for TaGSr-4B gene isolation in Westonia and Kauz, and its locations on the genome sequence

## Slide 4
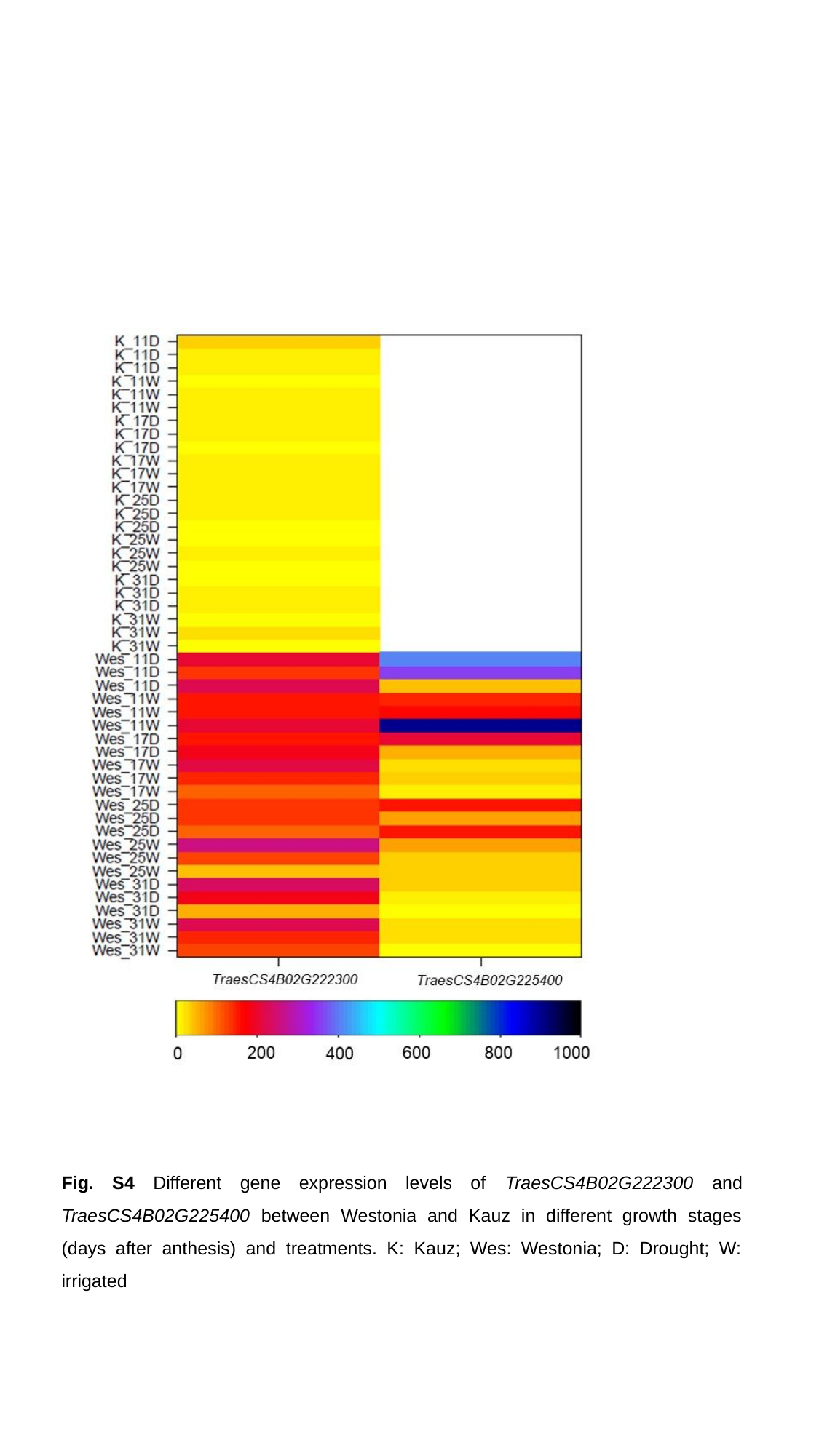

Fig. S4 Different gene expression levels of TraesCS4B02G222300 and TraesCS4B02G225400 between Westonia and Kauz in different growth stages (days after anthesis) and treatments. K: Kauz; Wes: Westonia; D: Drought; W: irrigated
